# Evolutionary loss of an antibiotic efflux pump increases *Pseudomonas aeruginosa* quorum sensing mediated virulence *in vivo*

**DOI:** 10.21203/rs.3.rs-5391023/v1

**Published:** 2024-11-12

**Authors:** Sheryl E. Fernandes, Humberto Ortega, Mylene Vaillancourt, Anna Clara M. Galdino, Aleksandr Stotland, Kyu Shik Mun, Diane Aguilar, Yohei Doi, Janet S. Lee, Elizabeth B. Burgener, Jeffrey E. Barrick, Jeffrey W. Schertzer, Peter Jorth

**Affiliations:** 1 Department of Pathology and Laboratory Medicine, Cedars-Sinai Medical Center, Los Angeles, CA, USA; 2 Binghamton Biofilm Research Center, Department of Biological Sciences, Binghamton University, Binghamton, NY, USA; 3 Smidt Heart Institute, Cedars-Sinai Medical Center, Los Angeles, CA, USA; 4 Board of Governor’s Regenerative Medicine Institute, Department of Medicine, Cedars-Sinai Medical Center, Los Angeles, CA, USA; 5 Department of Biomedical Sciences, Cedars-Sinai Medical Center, Los Angeles, CA, USA; 6 Center for Innovative Antimicrobial Therapy, Division of Infectious Diseases, Department of Medicine, University of Pittsburgh, Pittsburgh, PA, USA; 7 Division of Pulmonary and Critical Care Medicine, Department of Medicine, Washington University School of Medicine in St. Louis, St. Louis, MO, USA; 8 Department of Pediatrics, Division of Pulmonology, Children’s Hospital Los Angeles, Los Angeles, CA, USA; 9 Department of Pediatrics, Keck School of Medicine, University of Southern California, Los Angeles, CA, USA; 10 Department of Molecular Biosciences, Center for Systems and Synthetic Biology, The University of Texas at Austin, Austin, TX, USA

## Abstract

Antibiotic resistance is one of the most pressing threats to human health, yet recent work highlights how loss of resistance may also drive pathogenesis in some bacteria. In two recent studies, we found that β-lactam antibiotic and nutrient stresses faced during infection selected for the genetic inactivation of the *Pseudomonas aeruginosa* (*Pa*) antibiotic efflux pump *mexEFoprN*. Unexpectedly, efflux pump mutations increased *Pa* virulence during infection; however, neither the prevalence of efflux pump inactivating mutations in real human infections, nor the mechanisms driving increased virulence of efflux pump mutants are known. We hypothesized that human infection would select for efflux pump mutations that drive increased virulence in *Pa* clinical isolates. Using genome sequencing of hundreds of *Pa* clinical isolates, we show that *mexEFoprN* efflux pump inactivating mutations are enriched in *Pa* cystic fibrosis isolates relative to *Pa* intensive care unit clinical isolates. Combining RNA-seq, metabolomics, genetic approaches, and infection models we show that efflux pump mutants have elevated expression of two key *Pa* virulence factors, elastase and rhamnolipids, which increased *Pa* virulence and lung damage during both acute and chronic infections. Increased virulence factor production was driven by higher Pseudomonas quinolone signal levels in the efflux pump mutants. Finally, genetic restoration of the efflux pump in a representative ICU clinical isolate and the notorious CF *Pa* Liverpool epidemic strain reduced their virulence. Together, our findings suggest that mutations inactivating antibiotic resistance mechanisms could lead to greater patient mortality and morbidity.

## Introduction

The evolution of antibiotic resistance during chronic bacterial infections limits treatment options, increases healthcare costs, and is linked to worse outcomes in diseases like cystic fibrosis (CF) and in patients in the intensive care unit (ICU)^1–3^. However, there is also evidence of possible fitness costs associated with antibiotic resistance. Mutations that inactivate antibiotic resistance mechanisms might initially seem less favorable during bacterial pathogen evolution, but emerging evidence suggests the opposite: loss of some resistance functions can increase bacterial virulence and pathogenicity, and potentially worsen disease. This phenomenon is exemplified by our recent work showing that inactivating mutations of the *Pseudomonas aeruginosa* (*Pa*) *mexEFoprN* antibiotic efflux pump operon can increase virulence in an acute lung infection model^4,5^. Work from our lab and others have linked the occurrence of *mexEFoprN* inactivating mutations to increased resistance to aztreonam, cefiderocol, and imipenem-relebactam, which are not antibiotics transported by this efflux pump^5–7^. Despite these alarming data, questions remain regarding the prevalence of such mutations among real-world *Pa* clinical isolates as well as how these mutations function to increase *Pa* pathogenicity.

*Pa* is an opportunistic pathogen that constantly adapts and mutates under antibiotic pressure making *Pa* infections difficult to treat^8^. Hence, mechanisms of antibiotic resistance acquisition like overexpression of resistance/nodulation/division (RND) antimicrobial efflux pumps have been well studied^9^. MexEF-OprN is a tripartite *Pa* RND antibiotic efflux pump comprised of MexE, MexF, and OprN. The pump extrudes ciprofloxacin, quinolones, and chloramphenicol and inactivation of any component of the pump abrogates its efflux function. In the clinic, *Pa* isolates with activating mutations in the MexEF-OprN transcriptional activator, *mexT*^10^ or inactivating mutations in its transcriptional repressor *mexS*^11^ have been identified. These strains have historically been named *nfxC* mutants due to their norfloxacin resistance and have been used in several studies to characterize the functions of MexEF-OprN^10,12,13^.

In addition to increasing antibiotic resistance via overexpression of MexEF-OprN, *Pa nfxC* mutants have been associated with altered quorum sensing (QS) phenotypes. Since many *Pa* virulence factors are QS regulated^14^, the effect of MexEF-OprN on QS is expected to have consequences on *Pa* pathogenesis. One study demonstrated increased extracellular levels of the 3-oxo-C12-Homoserine Lactone (HSL) QS signaling molecule in stationary phase *nfxC* mutants, suggesting that 3-oxo-C12-HSL was a substrate of the efflux pump^15^. The 3-oxo-C12-HSL QS signal activates expression of the LasR-LasI QS system and its dependent virulence genes like *lasB,* which encodes elastase. Thus, the *nfxC* mutant had reduced activation of LasR-LasI and lower *lasB* expression^15^. However, other studies report increased levels of Pseudomonas Quinolone Signal (PQS) precursors HHQ^16^ or kynurenine^17^ in the supernatants of *nfxC* mutants, suggesting that MexEF-OprN overexpression reduces the biosynthesis of the PQS QS signal by pumping out its precursors. Consequently, this could reduce virulence of *nfxC* mutants because PQS activates biosynthesis of an arsenal of *Pa* pathogenic factors like elastase, rhamnolipid, pyocyanin and hydrogen cyanide^18,19^. It is however worth noting that *mexT* which is mutated in *nfxC* strains can regulate the expression of other *Pa* genes^20^ making it difficult to conclude if the reported effects on QS in *nfxC* mutants can be solely attributed to increased MexEF-OprN expression. Based on these conflicting findings relating *nfxC* mutants to altered QS phenotypes, questions remain: how do *mexEFoprN* inactivating mutations affect QS? And do *mexEFoprN* inactivating mutations increase *Pa* virulence in the host?

*Pa* is a major cause of acute respiratory infections in patients in the ICU ^2,21^ and chronic infections in people with CF (pwCF)^22^ and β-lactam antibiotics are the most widely used antibiotics to treat these infections^23,24^. Several studies have linked β-lactam antibiotic exposure and treatment to *mexEFoprN* inactivating mutations and increased virulence phenotypes. We have shown that either chronic aztreonam exposure or selection in synthetic CF sputum medium with or without the antibiotic cefiderocol can select for inactivating mutations in *mexEFoprN* during laboratory experimental evolution^5,6^. Several other reports suggest that *mexEFoprN* inactivating mutations are also selected *in vivo*. In *Pa* clinical isolates the increased expression of the β-lactam specific RND efflux pump, MexAB-OprM has been linked to reduced expression of MexEF-OprN^25^. Shields *et al.* found that imipenem-relebactam treatment led to inactivating mutations in either *mexE* or *mexF* in 4/5 critically ill ICU patients^7^. Notably, in the Shields *et al.* study, three patients expired after detection of *mexE* or *mexF* variants, suggesting a potential connection these variants and increased *Pa* virulence. Supporting the connection between *mexE* and *mexF* variants and *Pa* virulence, we showed that deletion of *mexEF* alone was sufficient to increase *Pa* virulence during acute murine infections^26^. We found that mutations of *mexE, mexF,* or *oprN* were sufficient to increase *Pa* surface swarming motility and expression of the *rhlA* gene involved in making the rhamnolipid surfactant that facilitates swarming^4^. These findings suggest that rhamnolipids may play a role in the increased virulence of *mexEFoprN* mutants. Despite these studies linking *mexEFoprN* inactivating mutations to increased *Pa* virulence, it is unclear if and how often *mexEFoprN* inactivating mutations arise during chronic infection and whether these mutations increase QS mediated virulence phenotypes *in vivo*.

In this study, we test the hypothesis that *mexEFoprN* inactivating mutations evolve during human infections and increase *Pa* virulence. Here, we determine the prevalence of *mexEFoprN* variants among ICU respiratory *Pa* isolates and CF *Pa* isolates. We then use bacterial genetics and infection models to elucidate how inactivation of MexEF-OprN affects lung infection and lung damage. Finally, we test whether these identified virulence mechanisms using representative ICU and CF *Pa* isolates with *mexEFoprN* inactivating mutations. Overall, this study highlights how mutations in antibiotic efflux pumps can have unexpected effects on *Pa* pathogenicity.

## Results

### Inactivating efflux pump mutations are enriched in CF clinical isolates

**Fig. 1:**
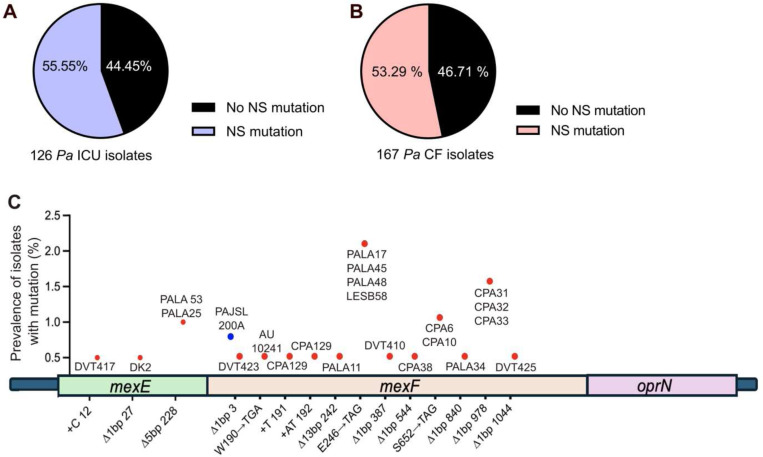
Inactivating mutations in *mexEFoprN* are enriched among CF *Pa* isolates. **a** Percentage of acute respiratory ICU *Pa* isolates with non-synonymous (NS) mutations in *mexE*, *mexF* or *oprN*. **b** Percentage of CF *Pa* isolates with NS mutations in *mexE*, *mexF,* or *oprN*. **c** Genomic location of *mexE* and *mexF* inactivating mutations in clinical isolates. Blue indicates prevalence of mutation among ICU *Pa* isolates and red indicates prevalence among CF *Pa* isolates.

We previously reported that chronic *in vitro* aztreonam exposure can select for deleterious mutations in the *Pa mexEFoprN* antibiotic efflux pump operon^5^ and others have detected *mexEFoprN* inactivating mutations in *Pa* ICU isolates^7^. To determine the relative prevalence of *mexEFoprN* inactivating mutations in *Pa* clinical isolates, genome sequences of 126 *Pa* isolates obtained from patients in the ICU and 167 isolates obtained from pwCF were analyzed. Using PAO1 as a reference genome, we found that 55.55% of ICU isolates and 53.29% of CF isolates had non-synonymous mutations in *mexE*, *mexF,* or *oprN* (Fig. 1A and 1B and Fig. S1A, S1B and S1C). Additionally, a *Pa* ICU isolate, PAJSL219A lacked the entire *mexEFoprN* operon and another, PAJSL200A had a frameshift mutation in *mexF* (Fig. 1C). Compared to ICU isolates, *mexE* or *mexF* inactivating mutations (deletions, nonsense mutations, and frameshift mutations) were enriched among *Pa* CF isolates (n=22/167 CF isolates vs. 2/126 ICU isolates, p=0.0002, Chi-square test) including the highly transmissible *Pa* Liverpool Epidemic Strain (LESB58) (Fig. 1C). Since all the components of the tripartite MexEF-OprN efflux pump are essential for its function, deleterious frameshift, nonsense, or deletion mutations in either *mexE* or *mexF* should inactivate the efflux pump in these clinical isolates.

### Efflux mutants cause lung barrier damage and increased inflammation during infection

**Fig. 2:**
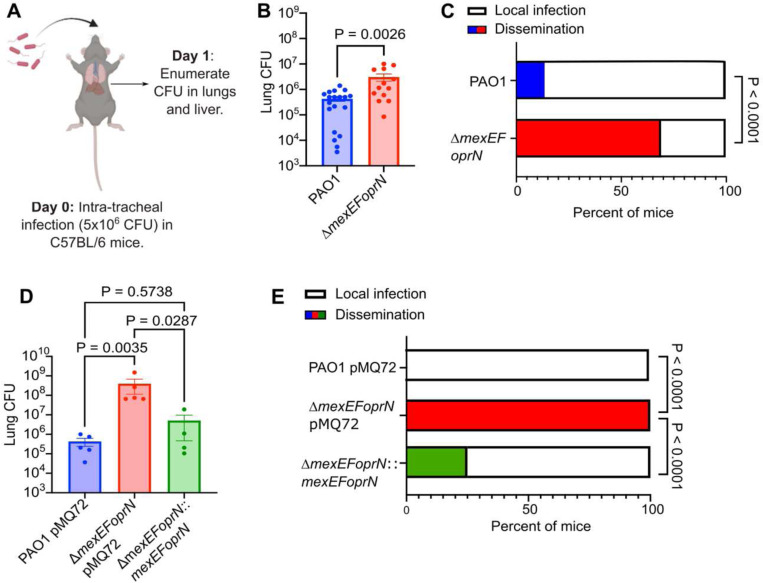
MexEF-OprN loss of function mutants cause lung barrier damage and increased inflammation during infection. **a** Schematic of acute lung infections in C57BL/6 mice. **b** Bacterial CFU enumerated in the lungs of PAO1 (n=21) or PAO1 Δ*mexEFoprN* (n=16) infected C57BL/6 mice at 24h post infection (hpi). Data show mean ± SEM. Statistical significance analyzed by unpaired t-test. **c** Number of PAO1 or PAO1 Δ*mexEFoprN* infected C57BL/6 mice with viable bacteria in the liver at 24 hpi. Statistical significance analyzed by Fisher’s exact test. **d** Bacterial CFU enumerated in the lungs of PAO1 pMQ72, PAO1 Δ*mexEFoprN* pMQ72 or PAO1 Δ*mexEFoprN* pMQ72::*mexEFoprN* infected C57BL/6 mice at 24hpi. Data show mean ± SEM. Statistical significance analyzed by ANOVA. **e** Number of PAO1 pMQ72 (n=5), PAO1 Δ*mexEFoprN* pMQ72 (n=5) or PAO1 Δ*mexEFoprN* pMQ72::*mexEFoprN* (n=4) infected C57BL/6 mice with viable bacteria in the liver at 24 hpi. Statistical significance analyzed by Fisher’s exact test.

Using an acute infection model with wild-type (WT) C57BL/6 mice (Fig. 2A), we previously found that PAO1 Δ*mexEFoprN* was hypervirulent relative to PAO1 WT, causing 90% mortality of mice at 48 h post-infection compared to 50% mortality of PAO1 WT infected mice at 96 h^21^. We sought to determine whether mortality was being driven by the bacteria directly or indirectly via the immune response to the bacterial infection. Bacterial burdens were quantified at 24 h post-infection in the lungs and livers of intratracheally infected mice. PAO1 Δ*mexEFoprN* infected C57BL/6 mice had ~10-fold higher lung bacterial burdens compared to PAO1 WT infected mice (Fig. 2B). Bacterial dissemination to the liver was also observed in 69% of PAO1 Δ*mexEFoprN* infected mice but only in 14% of PAO1 WT infected mice (Fig. 2C), thus indicating higher lung barrier damage and systemic infection in efflux pump mutant infected mice. Inflammatory cytokines were also upregulated in efflux pump mutant infected mice (Fig. S2), consistent with worse disease during mutant infections. Importantly, both bacterial replication in the lungs (Fig. 2D) and dissemination to the liver (Fig. 2E) could be reduced by complementation of the PAO1 Δ*mexEFoprN* mutant. These data showed that deletion of *mexEFoprN* contributes to PAO1 hypervirulence during acute infections by increasing bacterial burdens and lung inflammation.

### Increased elastase production drives efflux pump mutant virulence

Rhamnolipids (biosurfactant) and elastase (protease) are important *Pa* virulence factors that facilitate bacterial breakdown of the respiratory epithelial barrier by targeting intercellular tight junctions and degrading the host tissue matrix^27–29^. Previously we showed that mutations in *mexEFoprN* could increase swarming motility and related rhamnolipid gene expression of a rhamnolipid-GFP reporter construct *in vitro*^26^. Yet, it was unclear if rhamnolipid or other *Pa* virulence factors were driving the *mexEFoprN* efflux pump mutant’s increased virulence.

**Fig. 3:**
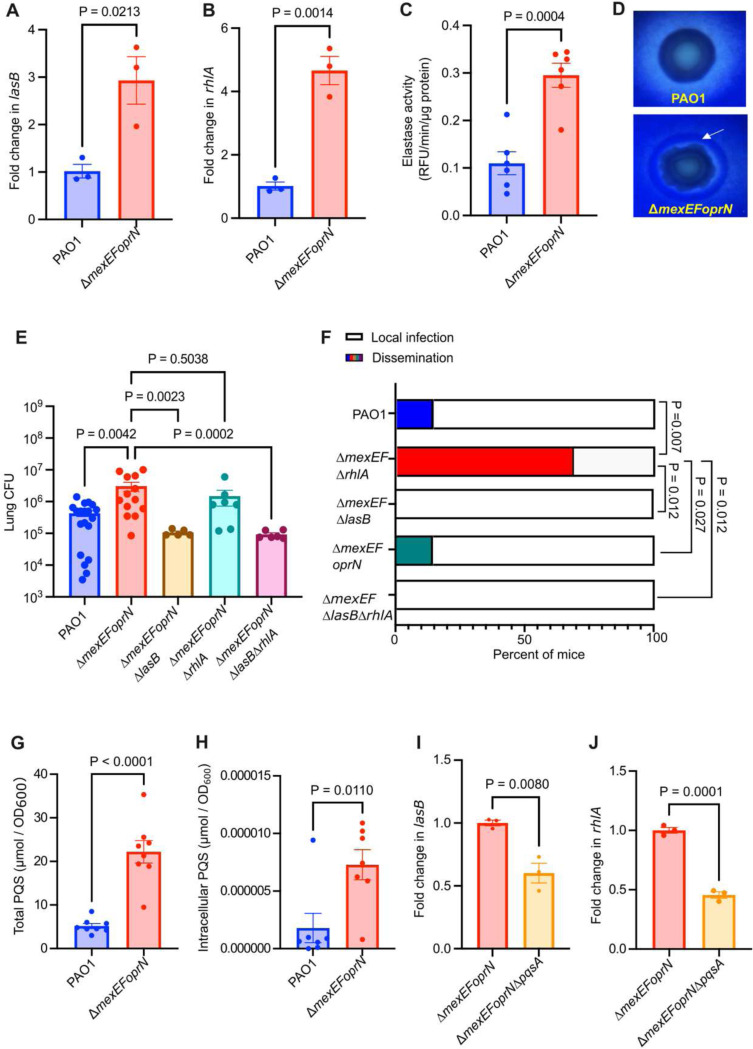
PQS dependent increase in levels of elastase and rhamnolipids drives hypervirulence of PAO1 Δ*mexEFoprN.* **a** Fold change in *lasB* gene expression in PAO1 and PAO1 Δ*mexEFoprN* measured by RT-qPCR. Data show mean ± SEM. Statistical significance analyzed by unpaired t-test. **b** Fold change in *rhlA* gene expression in PAO1 and PAO1 Δ*mexEFoprN* measured by RT-qPCR. Data show mean ± SEM. Statistical significance analyzed by unpaired t-test. **c** Elastase activity determined from the supernatants of PAO1 and PAO1 Δ*mexEFoprN* using a fluorogenic substrate. Fluorescence measured at 330 nm/460 nm (excitation/emission) is directly proportional to elastase activity and has been normalized to total protein levels in the culture supernatants. Total protein was quantified using Bicinchoninic acid (BCA). Data show mean ± SEM. Statistical significance analyzed by unpaired t-test. **d** Rhamnolipid production in PAO1 and PAO1 Δ*mexEFoprN* using cetyltrimethylammonium bromide (CTAB) methylene blue agar plates. A halo (white arrow) indicates rhamnolipid production. **e** Bacterial CFU enumerated in the lungs of PAO1, PAO1 Δ*mexEFoprN,* PAO1 *ΔmexEFoprNΔlasB,* PAO1 *ΔmexEFoprNΔrhlA* or PAO1 *ΔmexEFoprNΔlasB*Δ*rhlA* infected C57BL/6 mice at 24 hpi. Data show mean ± SEM. Statistical significance analyzed by ANOVA. **f** Percentage of PAO1 (n=21), PAO1 Δ*mexEFoprN* (n=16), PAO1 *ΔmexEFoprNΔlasB* (n=5), PAO1 *ΔmexEFoprNΔrhlA* (n=7) or PAO1 *ΔmexEFoprNΔlasB*Δ*rhlA* (n=6) infected C57BL/6 mice with viable bacteria in the liver at 24hpi. Statistical significance analyzed by Fisher’s exact test. **g** Total PQS levels quantified in PAO1 and PAO1 Δ*mexEFoprN* by thin-layer chromatography (TLC). Data show mean ± SEM. Statistical significance analyzed by unpaired t-test.**h** Intracellular PQS levels quantified in PAO1 and PAO1 Δ*mexEFoprN* quantified by TLC. Data show mean ± SEM. Statistical significance analyzed by unpaired t-test. **i** Fold change in *lasB* gene expression in PAO1 Δ*mexEFoprN* and PAO1 Δ*mexEFoprN*Δ*pqsA* measured by RT-qPCR. Data show mean ± SEM. Statistical significance analyzed by unpaired t-test. **j** Fold change in *rhlA* gene expression in PAO1 Δ*mexEFoprN* and PAO1 Δ*mexEFoprN*Δ*pqsA* measured by RT-qPCR. Data show mean ± SEM. Statistical significance analyzed by unpaired t-test.

First, we took a genome-wide approach to compare the transcriptome of the Δ*mexEFoprN* mutant to the WT strain. Transcriptomic analyses of the PAO1 Δ*mexEFoprN* efflux pump mutant relative to PAO1 WT grown in rich LB laboratory growth medium to mid-exponential phase (OD_600_ ~0.6) revealed few differences between the two strains. While the *mexE* and *mexF* genes were significantly downregulated in the mutant compared to WT as expected (Supplementary Table 1), among 10 up-regulated genes in the efflux pump mutant relative to PAO1 WT (>2-fold increase, adjusted p<0.05), only the *mvfR* gene, encoding the PQS-dependent response regulator, was consistent with our previous findings (Supplementary Table 1). This was surprising, since we previously detected increased expression of the rhamnolipid biosynthesis gene, *rhlA* in the efflux pump mutant relative to WT^4^. However, since the RNA-seq analyses were performed at a single time point in mid-exponential phase growth (OD600 ~0.6), and MvfR can increase rhamnolipid gene expression^18^, we sought to quantify the expression of rhamnolipid (*rhlA*) and another *mvfR*-regulated virulence factor, elastase (*lasB*), at a later growth phase when QS genes typically become induced (early stationary phase; OD600 ~1.0). In early stationary phase, *lasB* and *rhlA* gene expression were analyzed by qRT-PCR and both were increased ~3-fold and ~4-fold, respectively, in the PAO1 Δ*mexEFoprN* efflux pump mutant compared to PAO1 WT (Fig. 3A and 3B). Complementation of PAO1 Δ*mexEFoprN* reduced elastase and *rhlA* gene expression (Fig. S3A and S3B). Phenotypic analyses were consistent with gene expression, as elastase and rhamnolipid production were increased in the mutant using a fluorogenic elastase reporter substrate (Fig. 3C) and the rhamnolipid plate assay (Fig. 3D), respectively.

To investigate if increased levels of elastase and rhamnolipids were driving hypervirulence of PAO1 Δ*mexEFoprN*, we constructed *lasB* or *rhlA* deletion mutants in the PAO1 Δ*mexEFoprN* mutant background and performed acute infections in C57BL/6 mice. Eliminating elastase biosynthesis reduced bacterial lung burdens 100-fold but eliminating rhamnolipid biosynthesis did not affect lung burdens (Fig. 3E). However, significantly fewer mice infected with PAO1 Δ*mexEFoprN*Δ*lasB* or PAO1 Δ*mexEFoprN*Δ*rhlA* showed lung barrier damage and bacterial dissemination to the liver than the PAO1 Δ*mexEFoprN* mutant, indicating that both elastase and rhamnolipids contribute to breaching the lung epithelial barrier (Fig. 3F). Increased expression of inflammatory genes in the lungs of PAO1 Δ*mexEFoprN* infected mice also indicated more severe lung damage compared to PAO1 Δ*mexEFoprN*Δ*lasB* and PAO1 Δ*mexEFoprN*Δ*rhlA* infected mice (Fig. S2). Further, deletion of both *lasB* and *rhlA* did not have an additive effect on lung infection as bacterial lung burdens of the PAO1 Δ*mexEFoprN*Δ*lasB*Δ*rhlA* triple mutant were equivalent to the PAO1 Δ*mexEFoprN*Δ*lasB* double mutant (Fig. 3E), and lung barrier damage was also eliminated in both the PAO1 Δ*mexEFoprN*Δ*lasB*Δ*rhlA* triple mutant and PAO1 Δ*mexEFoprN*Δ*lasB* double mutant (Fig. 3F).

### Increased PQS levels drive virulence of efflux pump mutants

In addition to elastase and rhamnolipid biosynthesis genes being regulated by MvfR, both virulence factors are regulated by two other *Pa* quorum sensing (QS) systems. Specifically, the elastase gene *lasB* is regulated by the LasR-LasI QS system and the 3-oxo-C12-HSL QS signal^30^ and the rhamnolipid gene *rhlA* is regulated by the RhlR-RhlI QS system and the C4-HSL QS signal^30^. As noted above, both can also be regulated by the third QS system where PQS is the signaling molecule that binds the transcription factor MvfR increasing *lasB* and *rhlA* gene expression^18,19^. Previous studies have associated increased *mexEFoprN* expression with changes in QS signals and precursors, but have conflicted. Among these studies, increased *mexEFoprN* expression has been linked to increased extracellular levels of 3-oxo-C12-HSL and the PQS precursor molecules HHQ and kynurenine^15–17^. Additionally, increased *mexEFoprN* expression has been associated with reduced expression of 3-oxo-C12-HSL-dependent and PQS-dependent genes^15–17^. Based on these complicated previous results, we next wanted to determine if deletion of MexEF-OprN in the mutant could affect the intracellular levels of 3-oxo-C12-HSL, PQS, or PQS precursors and explain increased elastase and rhamnolipid biosynthesis.

Metabolomic analyses were performed on PAO1 Δ*mexEFoprN* mutant and PAO1 WT cells and supernatants. Intracellular and extracellular PQS levels were higher in the PAO1 Δ*mexEFoprN* strain compared to PAO1 WT (Fig. S4C and S4F), while neither intracellular nor extracellular levels of 3-oxo-C12-HSL or C4-HSL were significantly different (Fig. S4A, S4B, S4D and S4E). The intracellular level of the PQS precursor kynurenine was notably more abundant in the PAO1 Δ*mexEFoprN* strain compared to PAO1 WT but complementation of PAO1 Δ*mexEFoprN* did not reduce intracellular kynurenine to WT levels (Fig. S4G). Together with our previous findings from *in vivo* and complementation studies (Fig. 2), these metabolomic data suggest that increased levels of intracellular kynurenine are not the driving factor for the mutant’s increased virulence.

To further test effects of loss of *mexEFoprN* on PQS signaling, both total (Fig. 3G) and intracellular PQS (Fig. 3H) were quantified by thin layer chromatography and PQS was more abundant in PAO1 Δ*mexEFoprN* compared to PAO1 WT and the complemented mutant strain (Fig. S4H–I). To test whether increased *lasB* and *rhlA* expression was dependent on PQS, we generated a PAO1 Δ*mexEFoprN*Δ*pqsA* strain and determined the expression of these genes in the generated mutant strain. Deletion of *pqsA* in the Δ*mexEFoprN* mutant background resulted in a 2-fold reduction in *lasB* and *rhlA* expression (Fig. 3I and 3J). Because intracellular levels of the PQS precursor kynurenine were also higher in PAO1 Δ*mexEFoprN* compared to PAO1 WT, we hypothesized that increased PQS production and subsequent elastase and rhamnolipid gene expression could be dependent on kynurenine biosynthesis. To test this hypothesis, we deleted the kynurenine biosynthesis gene *kynU* in the PAO1 Δ*mexEFoprN* background and measured PQS levels and *lasB* and *rhlA* gene expression in this strain. Unexpectedly, the PAO1 Δ*mexEFoprN* and PAO1 Δ*mexEFoprN*Δ*kynU* mutants produced equal levels of PQS, with equivalent *lasB* and *rhlA* gene expression (Fig. S5), confirming that accumulation of intracellular kynurenine was not responsible for increased PQS levels and *rhlA* or *lasB* gene expression in the efflux pump mutant.

### *Pa* efflux mutants cause increased damage in CF infection models

Genomic analyses showed that *mexEFoprN* inactivating mutations were enriched in CF clinical isolates relative to ICU isolates, leading us to hypothesize that these mutations would be beneficial to *Pa* during CF infection. To test this hypothesis, we used a human CF airway epithelial cell infection model and a transgenic mouse infection model that mimics mucus and inflammatory signatures of human CF infection^31–33^.

**Fig. 4:**
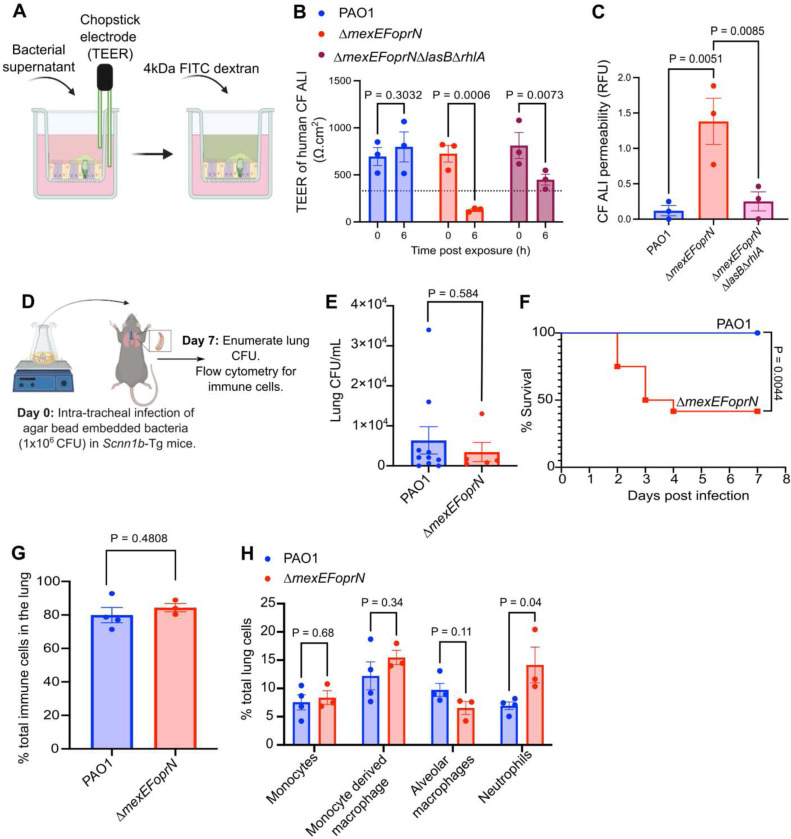
Hypervirulence of PAO1 Δ*mexEFoprN* is due to increased levels of elastase and rhamnolipids. **a** Schematic of CF lung barrier dysfunction assay. **b** Transepithelial electrical resistance (TEER) of air liquid interface (ALI) cultures derived from human cystic fibrosis (CF) airways (ΔF508/ΔF508) measured using STX2 chopstick electrodes. TEER < 330 Ω.cm^2^ (dotted line) indicates a loss in epithelial barrier function. TEER was recorded before (0h) and at 6h after exposure to PAO1, PAO1 Δ*mexEFoprN* or PAO1 *ΔmexEFoprNΔlasB*Δ*rhlA* supernatants. Data show mean ± SEM. Statistical significance analyzed by ANOVA. **c** Permeability of the CF airway epithelial barrier determined using 4kDa fluorescein isothiocyanate (FITC) labelled dextran at 6h post exposure to PAO1, PAO1 Δ*mexEFoprN* or PAO1 *ΔmexEFoprNΔlasB*Δ*rhlA* supernatants. Data show mean ± SEM. Statistical significance analyzed by ANOVA. **d** Schematic of chronic lung infections in *Scnn1b*-Tg mice. **e** Bacterial CFU enumerated on day 7 from the lung homogenates of *Scnn1b*-Tg mice infected with PAO1 or PAO1 Δ*mexEFoprN* embedded in agar beads. Data show mean ± SEM. Statistical significance analyzed by unpaired t-test. **f** Survival curves of *Scnn1b*-Tg mice infected with PAO1 (n=10) or PAO1 Δ*mexEFoprN* (n=13) embedded in agar beads. Statistical significance analyzed by Mantel-Cox test. **g-h** Flow cytometry for total immune cells, monocyte, macrophages and neutrophils in the lungs of PAO1 or PAO1 Δ*mexEFoprN* infected mice at day 7 post infection. Data show mean ± SEM. Statistical significance analyzed by unpaired t-test.

Air liquid interface (ALI) cultures derived from human CF (ΔF508/ΔF508 *CFTR*) airway progenitor cells were treated with the secreted products in supernatants from PAO1, PAO1 Δ*mexEFoprN*, and PAO1 Δ*mexEFoprN*Δ*lasB*Δ*rhlA* cultures to test whether increased levels of elastase and rhamnolipids in the efflux pump mutant cause epithelial barrier damage. CF airway damage was quantified by measuring the transepithelial electrical resistance (TEER) of CF cultures before (0h) and after exposure to bacterial culture supernatants until the TEER decreased below 330Ω.cm^2^, indicating a loss in barrier integrity (Fig. 4A). At 6h post exposure, the TEER of CF cultures exposed to PAO1 Δ*mexEFoprN* supernatant was lower than 330Ω.cm^2^ (Fig. 4B), indicating loss of epithelial barrier function. However, no loss of barrier function was observed in CF epithelia exposed to PAO1 or PAO1 Δ*mexEFoprN*Δ*lasB*Δ*rhlA* supernatants (Fig. 4B). Epithelial barrier permeability was also assessed using Fluorescein isothiocyanate (FITC) labelled 4kDa dextran (Fig. 4A). At 6 h, only CF epithelia exposed to PAO1 Δ*mexEFoprN* supernatants showed increased dextran permeability (Fig. 4C). Together, these data indicate that increased elastase and rhamnolipids in PAO1 Δ*mexEFoprN* can cause damage to the CF airway epithelium.

Next, we tested the virulence of PAO1 Δ*mexEFoprN* using a low dose chronic lung infection model in *Scnn1b*-Tg mice. The *Scnn1b*-Tg mice overexpress the epithelial sodium channel resulting in airway mucus accumulation, reduced mucociliary clearance, and neutrophil infiltration similar to lung airways in pwCF^32^. *Scnn1b*-Tg mice were infected with a low dose (1×10^6^ colony forming units [CFUs]) of PAO1 WT or PAO1 Δ*mexEFoprN* embedded in SCFM2 agar beads (Fig. 4D). Using SCFM2 makes the model more CF-like because *Pa* gene expression patterns in SCFM2 mirror *Pa* gene expression patterns in CF sputum^34,35^. Although we did not observe a difference in the lung burden of PAO1 WT or PAO1 Δ*mexEFoprN* infected mice that survived through day 7 (Fig. 4E), the mutant caused lethality of 50% infected mice by day 3 post infection, but no lethality was observed in PAO1 infected mice (Fig. 4F). Moreover, in the surviving mutant infected mice, there were significantly more neutrophils in the lungs compared to WT PAO1 infected mice (Fig. 4G and 4H). This indicates that PAO1 Δ*mexEFoprN* is hypervirulent and causes increased lung inflammation compared to PAO1 during chronic infections in *Scnn1b*-Tg mice.

### Restoration of *mexEFoprN* reduces virulence of *Pa* clinical isolates

So far, our findings using the laboratory strain PAO1 indicate that inactivation of *mexEFoprN* was sufficient to increase *Pa* virulence through increased elastase and rhamnolipids. Because strain PAO1 has a *mexS* mutation that affects *mexEFoprN* expression^36^, we wanted to test effects of *mexEFoprN* inactivating mutations in other strain backgrounds and on the virulence of *Pa* clinical isolates. For this, we genetically restored wild-type copies of the *mexEFoprN* operon in *Pa* PAJSL219A, an acute respiratory ICU isolate that lacked the *mexEFoprN* operon and *Pa* LESB58, a CF isolate with a nonsense mutation in *mexF*. Since *lasB* and *rhlA* were important virulence factors in the PAO1 Δ*mexEFoprN* mutant, we hypothesized that the *mexEFoprN* mutations in the two clinical isolates would also increase expression of these important virulence factors.

**Fig. 5:**
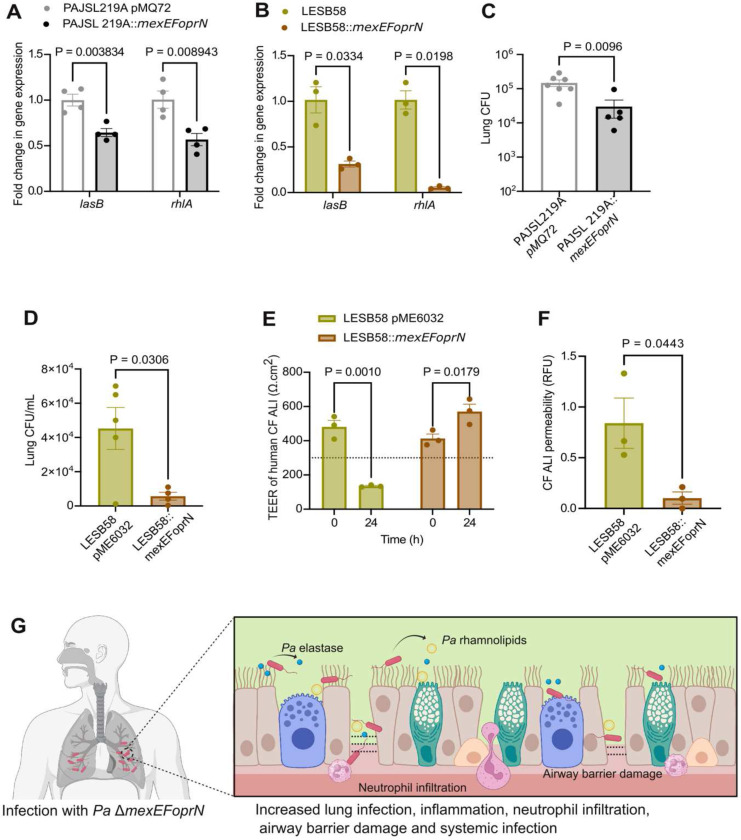
Restoration of *mexEFoprN* expression reduces virulence of clinical *Pa* isolates. **a** Fold change in *lasB* and *rhlA* gene expression in PAJSL219A pMQ72 and PAJSL219A pMQ72::*mexEFoprN* measured by RT-qPCR. Statistical significance analyzed by unpaired t-test. **b** Fold change in *lasB* and *rhlA* gene expression in LESB58 pME6032 and LESB58::*mexEFoprN* measured by RT-qPCR. Statistical significance analyzed by unpaired t-test. **c** Bacterial CFU were enumerated in the lungs of PAJSL219A pMQ72 and PAJSL219A::*mexEFoprN* infected C57BL/6 mice at 24 hpi. Statistical significance analyzed by unpaired t-test. **d** Bacterial CFU enumerated on day 7 from the lung homogenates of *Scnn1b*-Tg mice infected with *Pa* LESB58 pME6032 or *Pa* LESB58 pME6032::*mexEFoprN* embedded in agar beads. Statistical significance analyzed by unpaired t-test. **e** Transepithelial electrical resistance (TEER) of air liquid interface (ALI) cultures derived from human cystic fibrosis (CF) airways (ΔF508/ΔF508) measured using STX2 chopstick electrodes. TEER < 330 Ω.cm^2^ (dotted line) indicates a loss in epithelial barrier function. TEER was recorded before (0h) and at 24h after exposure LESB58 pME6032 or LESB58::*mexEFoprN* supernatants. TEER < 330 Ω.cm^2^ (dotted line) indicates a loss in epithelial barrier function. Statistical significance analyzed by ANOVA. **f** Permeability of the CF airway epithelial barrier determined using 4kDa fluorescein isothiocyanate (FITC) labelled dextran at 24h post exposure to LESB58 pME6032 or LESB58::*mexEFoprN* supernatants. Statistical significance analyzed by unpaired t-test. **g** Model depicting the important virulence mechanisms enhanced in *Pa* Δ*mexEFoprN* and the effect on the host during infection. Efflux pump mutants increase elastase and rhamnolipid production, leading to increased epithelial damage, replication during infection, dissemination, and lethality *in vivo*. In a-f, data shown are mean ± SEM.

We analyzed *lasB* and *rhlA* gene expression in the complemented clinical isolates by RT-qPCR. As predicted, restoration of the WT *mexEFoprN* operon in both *Pa* PAJSL219A and *Pa* LESB58 reduced *lasB* and *rhlA* gene expression (Fig. 5A and 5B).

Because *Pa* PAJSL219A is an acute respiratory infection ICU isolate, acute infections of C57BL/6 mice were carried out with *Pa* PAJSL219A pMQ72 (vector control) and *Pa* PAJSL219A pMQ72::*mexEFoprN* (restored strain). Restoration of the wild-type *mexEFoprN* operon resulted in ~10-fold decrease in the lung bacterial burden of *Pa* PAJSL219A pMQ72::*mexEFoprN* infected mice compared to *Pa* PAJSL219A pMQ72 infections (Fig. 5C). However, no bacterial dissemination was observed in either *Pa* PAJSL219A pMQ72 or *Pa* PAJSL219A pMQ72::*mexEFoprN* infected mice, demonstrating that invasiveness varies in different *Pa* strain backgrounds.

To test if introducing a wild-type copy of the *mexEFoprN* operon would also reduce the virulence of the CF isolate *Pa* LESB58, *Scnn1b*-Tg mice were infected with *Pa* LESB58 pME6032 (vector control) or *Pa* LESB58 pME6032::*mexEFoprN* (restored strain). On day 7 post-infection, the lung bacterial burden in *Pa* LESB58 pME6032 infected mice was 4-fold higher than *Pa* LESB58 pME6032::*mexEFoprN* infected mice (Fig. 5D). To test if the *mexEFoprN* inactivating mutation in LESB58 increased CF airway damage, CF human lung epithelial cultures were treated with supernatants from *Pa* LESB58 pME6032 (vector control) or *Pa* LESB58 pME6032::*mexEFoprN* (restored strain). Epithelial damage was determined by reduced TEER and increased dextran permeability. At 24h, CF epithelial cultures exposed to *Pa* LESB58 pME6032 vector control supernatant showed a significant reduction in barrier integrity (Fig. 5E) and increased dextran permeability (Fig. 5F) but the integrity of CF epithelial cultures exposed to the supernatant of *Pa* LESB58 pME6032::*mexEFoprN* restored strain was not compromised (Fig. 5E and 5F). These data indicate that *mexEFoprN* inactivating mutations contribute to the increased virulence of *Pa* clinical isolates, including abilities to infect and damage lung epithelial barriers.

## Discussion

*Pa* infects greater than 60% of adults with CF^22^ and is one of the most common causes of acute respiratory infections in patients in the ICU. In both pwCF and patients in the ICU, *Pa* infections are associated with lung function decline and increased morbidity^2,37–39^. Hence, identifying mutations that contribute to *Pa* hypervirulence is important. Here, we demonstrate that inactivating mutations in the *mexEFoprN* operon which were identified in ~2% of ICU *Pa* isolates and ~13.17% of CF *Pa* isolates analyzed in this study can increase the virulence of *Pa* during chronic CF infections and in acute lung infections. While it is not known if any host factors select for these mutations, prolonged β-lactam exposure can select for *mexEFoprN* inactivating mutations^5,7^, which is concerning because β-lactam antibiotics are used to treat both chronic CF *Pa* infections and acute respiratory *Pa* infections.

Increased *Pa* elastase activity correlates with a higher 30-day mortality in *Pa* infected patients in the ICU^40^. In the acute murine infection model, we observed increased lethality, increased systemic infection, and pulmonary inflammation in PAO1 Δ*mexEFoprN* infected mice which was associated with elevated elastase and rhamnolipid levels. This indicates that patients infected with *Pa* Δ*mexEFoprN* strains maybe at a higher risk of sepsis, morbidity, and mortality.

While the ICU isolate PAJSL219A lacked the *mexEF-oprN* operon, it did not show increased dissemination relative to PAO1. At first, this result may seem unexpected; however, it is well appreciated that virulence can vary greatly from one *Pa* strain to another^41^. Thus, it is perhaps unsurprising that lung barrier damage was not observed during acute infections with the PAJSL219A because the presence of other genomic mutations could be responsible for reduced dissemination relative to the PAO1 genomic background. Consistent with our experiments with the PAO1 Δ*mexEFoprN* mutant, restoring a wild-type copy of *mexEFoprN* in PAJSL219A reduced its elastase and rhamnolipid expression and lung bacterial burden in infected mice. These data indicate that despite other confounding factors, the *mexEFoprN* mutation increased virulence factor expression of PAJSL219A.

We observed that restoring *mexEFoprN* in the CF isolate *Pa* LESB58 reduced elastase and rhamnolipid expression and human CF airway damage. Unlike PAO1 Δ*mexEFoprN* chronic infections, we did not see any lethality of *Scnn1b*-Tg mice infected with LESB58. This could again be due to other genomic mutations in LESB58 relative to PAO1, but restoring *mexEFoprN* expression in the strain resulted in reduced lung bacterial burdens which is consistent with our PAO1 findings. LES was the first CF epidemic strain identified having infected 79% of pwCF in a Liverpool adult CF center^42^. Besides the UK, the LES strain has been detected in North America^43^, highlighting its high transmissibility. Further, LESB58 infections have been associated with pulmonary and extrapulmonary exacerbations in pwCF^44–46^, and LES was associated with higher 3 year mortality in Canadian pwCF compared to pwCF in Canada infected with other *Pa* strains^47^. While *Pa* LESB58 is undoubtedly an aggressive strain, its mechanisms of enhanced virulence remain unclear^48^. In this study, we demonstrate that the virulence of LESB58 could be driven by *mexF* inactivation leading to increased elastase and rhamnolipids. Since few strains are as readily transmissible as LES, it is tempting to hypothesize that the *mexF* mutation may also contribute to its transmissibility from person-to-person. Unfortunately, while infection transmission models are frequently used for viruses like influenza^49^, such models do not yet exist for *Pa.* Adapting transmission models for *Pa* analyses could help address such questions in future studies. Alternatively, it would be interesting to examine other epidemic clones of *Pa*^50,51^ in CF and other infection types to determine whether *mexEFoprN* mutations are more common among transmissible lineages of *Pa* compared to *Pa* that do not spread from person-to-person.

Highly effective modulator therapies (HEMT) that correct cystic fibrosis transmembrane regulator (CFTR) dysfunction have been a breakthrough in the management of CF disease, improving outcomes and prolonging life. As HEMT reduce airway mucus accumulation which makes pwCF susceptible to chronic *Pa* infections, the rate of *Pa* detection in CF sputum by traditional culture has decreased^52^. However, using sensitive sequencing-based detection methods, several studies reveal that *Pa* infections generally persist following HEMT^53–56^. Notably, in the study by Ledger *et al*, 3 pwCF who were infected with *mexE* and *mexF* variants received HEMT yet had persistent lung colonization by these variants even up to 12 months post-HEMT initiation, indicating that these strains are difficult to eradicate^56^. While it is unknown if mutations in *mexE* and *mexF* may play a role in adaptation to the CF airways, our findings suggest that persistent infections with these strains could result in severe lung damage.

It is also important to note several limitations that affect our conclusions. Our analyses of representative clinical isolates suggest that inactivating mutations in *mexEFoprN* could have widespread effects in other clinical isolates. However, the effects of missense mutations in *mexEFoprN* that are found in ~50% of *Pa* clinical isolates have yet to be tested. Likewise, our findings using the PAO1 strain indicate that increased elastase and rhamnolipid expression was PQS dependent in the deletion mutant; however, this is yet to be tested in clinical isolates. It is possible that secondary mutations affecting the biosynthesis of PQS, elastase, or rhamnolipids could prevent *mexEFoprN* mutations from increasing virulence in those strains. Thus, it will be important to understand how *mexEFoprN* mutations are affected by epistatic interactions with other *Pa* mutations. Another limitation to our research is that many of the *in vitro* experiments, including transcriptomic and metabolomic experiments were performed in LB broth and it is possible that the nutritional environment and other stresses present during infection could affect the signaling pathways involved in PQS upregulation in the mutant. Future studies will address these open questions through metabolomic analyses and dual-RNA-seq analyses of infected animals.

Finally, *Pa* has 12 RND antibiotic efflux pumps and the antimicrobial substrates of these pumps have been well characterized^9^. However, many of these pumps are also expressed in the absence of antibiotics indicating they may have functions beyond antibiotic efflux. Although identification of alternative non-antibiotic substrates of MexEF-OprN was beyond the scope of this study, we use multiple infection models to show that mutations leading to the loss of MexEF-OprN function increases *Pa* virulence (Fig. 5G). Our findings also caution against the clinical use of broad-spectrum efflux pump inhibitors, which are currently in clinical development, to tackle *Pa* antimicrobial resistance as inhibition of some efflux pumps like MexEF-OprN could increase *Pa* pathogenesis.

## Methods

### Animals

C57BL/6 and *Scnn1b-Tg* mice were obtained from The Jackson Laboratory. Mice were 8–12 weeks old at the time of experiments. Animal protocols were approved by the Cedars-Sinai Institutional Animal Care and Use Committee (IACUC protocols #8115).

### Bacterial strains and growth conditions

Bacterial strains and plasmids are listed in Table S2. Deletion mutants were derived from *P*. *aeruginosa* PAO1, which was obtained from Colin Manoil’s laboratory (University of Washington, USA). All strains were grown at 37°C in lysogeny broth (LB) or LB agar (Becton, Dickinson (BD) Biosciences, USA), unless otherwise specified. When necessary, mutants were selected by adding the following antibiotics to the growth medium: gentamicin (Gm): 10 μg/mL for *E*. *coli* and 30 μg/mL for *P*. *aeruginosa*; tetracycline: 10 μg/mL for *E*. *coli* and 200 μg/mL for *P. aeruginosa* LESB58.

### Mutant construction

Gene deletion mutants were generated using the suicide plasmid vector, pEX18Gm as described previously^57^. Briefly, ~1 kb of the upstream and downstream regions of each gene were amplified from the PAO1 chromosomal DNA (primers specified in table S3). The amplified upstream and downstream fragments were then assembled into pEX18Gm using NEBuilder HiFi DNA Assembly Cloning Kit (New England Biolabs, USA). The pEX18Gm plasmids were transformed into *E. coli* DH5α as specified in the NEB protocol and later verified by Sanger sequencing. *P. aeruginosa* strains were transformed with the verified pEX18Gm plasmids by electroporation followed by selection on LB plates containing Gm 30 μg/mL at 37°C overnight. Isolated clones were then counter selected on no sodium LB plates containing 15% sucrose at room temperature (RT) for 48h. Deletion mutants were confirmed by PCR and whole genome sequencing.

To complement PAO1 Δ*mexEFoprN* and the clinical isolates 129A and *Pa* LESB58, the entire *mexEFoprN* operon was amplified from PAO1 chromosomal DNA using primers specified in Table S2. Amplified fragments were assembled into the expression vectors pMQ72 or pME6032 (NovoPro Bioscience, China) using NEBuilder HiFi DNA Assembly Cloning Kit (New England Biolabs, USA) and transformed into *E. coli* DH5α as specified in the NEB protocol. After verification by Sanger sequencing, *P. aeruginosa* strains were transformed with the plasmids by electroporation and mutants were selected on LB plates containing gentamicin (pMQ72) or tetracycline (pME6032).

### DNA extraction, purification and PCR

Plasmid DNA was isolated using Monarch Plasmid Miniprep Kit (New England Biolabs, USA). Genomic DNA was isolated using the DNeasy Blood & Tissue Kit (Qiagen, Germany). PCR was performed using either KAPA HIFI 2X ready mix (KAPA Biosystem, USA). Primers used are listed in Table S2.

### Genome analysis of *Pseudomonas aeruginosa* clinical isolates

*P*. *aeruginosa* ICU respiratory strains were a generous gift from the Pulmonary Translational Research Core at University of Pittsburgh. Genomic DNA was isolated from these strains using the DNeasy Blood & Tissue Kit (Qiagen, Germany) and were sequenced by Microbial Genome Sequencing Center (MiGs, Pittsburgh, USA).

Genome sequences of 98 *P. aeruginosa* CF strains were downloaded from GenBank. The remaining genome sequences came from *Pa* isolated from CF sputum at the Stanford CF Center. The nucleotide sequences of all strains were systematically sampled at every position and on each strand to create 200-bp sequencing reads tiling the entire genome. Variants were called, annotated, and compared by examining how these simulated read datasets aligned to the *P. aeruginosa* PAO1 reference genome. The *breseq* pipeline^58^ (v0.38.3) and its associated *gdtools* utility program were used to conduct this analysis.

### RNA-seq analyses

Overnight LB cultures of PAO1 WT and PAO1 Δ*mexEFoprN* were diluted in LB broth and grown at 37°C and shaking at 250 RPM until cultures reached a density of OD_600_ ~0.6. Cultures were then centrifuged at 12,000×g for 5 minutes and cell pellets were resuspended in RNA later and stored in –80°C until RNA extraction. RNA was isolated using the RNeasy mini-Kit (Qiagen, Germany) and residual DNA was removed using a DNA-free DNA Removal Kit (Invitrogen, USA). Ribosomal RNA was depleted using Ribo-Zero rRNA Removal Kit (Illumina). RNA sequencing libraries were prepared using the Illumina Stranded Total RNA Prep kit (Illumina Inc., USA) and sequenced using an Illumina NovaSeq6000 at the Cedars-Sinai Cancer Applied Genomics Core targeting 30M reads per sample. RNA-seq reads were trimmed to remove adapter sequences using Flexbar^59^. Trimmed reads were aligned to the PAO1 reference genome using Bowtie and samtools^60,61^. Raw read counts per PAO1 gene were calculated using HT-Seq^62^. Read counts per gene were normalized and differential gene expression was calculated using DESeq2^63^.

### RNA isolation and qRT-PCR

To determine the expression of *lasB* and *rhlA*, overnight LB cultures were diluted in fresh LB and incubated at 37°C and shaking at 250 RPM till cultures attained OD600 of ~1.0. RNA was isolated using the RNeasy mini-Kit (Qiagen, Germany) and residual DNA was removed using a DNA-free DNA Removal Kit (Invitrogen, USA). SuperScript III First-Strand Synthesis SuperMix (Invitrogen, USA) was used to synthesize cDNA and PowerUp SYBR Green master mix (Applied Biosystems, USA) was used for qPCR. The primers used are listed in Table S2. *rpoD* was used as the housekeeping gene and gene expression was calculated using the 2^−ΔΔCt^ method.

### Elastase activity measurement

To determine the levels of secreted elastase, bacterial strains were incubated overnight in LB at 37°C and shaking at 250RPM. Cell free supernatants were then obtained by filtering the overnight culture through 0.22-μm syringe filters. After estimating the total protein amounts in the supernatants using bicinchoninic acid (BCA) assay kit (Thermo Fisher Scientific, USA), supernatants were diluted in assay buffer (10 mM of Tris-HCl pH 7.2, 10 mM CaCl2). In a single well of a 96-well clear bottom black plate, 10 μL of diluted supernatant (containing 20–50 μg total protein) was mixed with 90 μL assay buffer containing 200 μM Abz-Ala-Gly-Leu-Ala-p-Nitro-benzyl-amide (Biosynth Ltd., UK). All the reactions were prepared on ice. Fluorescence (Ex: 330 nm, Em: 460 nm) from the wells was then measured in a kinetic mode every minute for 30 minutes using a Varioskan Lux Microplate Reader (Thermo Fisher Scientific, USA). Elastase activity (RFU/min/μg protein) was determined as a ratio of the slope obtained from the curve plotted between fluorescence and time (RFU/min) and the total protein amount (μg) in each sample.

### Rhamnolipid plate assay

Rhamnolipid plates were prepared according to a previously described protocol^64^ with some modifications in the medium composition. The rhamnolipid plates were prepared with M9 salts (BD Biosciences, USA), 0.2% glucose, 0.02% methylene blue, 0.05% cetyltrimethylammonium bromide, and 1.5% agar. Overnight LB cultures of bacterial strains grown at 37°C and shaking at 250 RPM were then spotted onto the rhamnolipid plates. Plates were incubated at 37°C for 24 h and then at RT until the appearance of a blue halo, indicating the production of rhamnolipids.

### Metabolomics analyses

PAO1 pMQ72, PAO1 Δ*mexEFoprN* pMQ72 and PAO1 Δ*mexEFoprN:mexEFoprN* cultures were grown in LB broth to OD_600_ of 0.8. Cultures were centrifuged at 4,000xg for 15 minutes and the supernatant was separated from the cell pellet. The cell pellet was washed in PBS, flash frozen and then stored in –80°C till metabolite extraction. The culture supernatant was filtered through a 0.22-μm filter to remove any remaining live bacteria, flash frozen and stored in –80°C until metabolite extraction. Metabolite extraction and metabolomic analysis was performed at the UCLA metabolomics core. Briefly, dried metabolites were resuspended in 50 μL 50% ACN:water and 5 μL was loaded onto a Luna NH_2_ 3μm 100A (150 × 2.0 mm) column (Phenomenex) using a Vanquish Flex UPLC (Thermo Scientific). The chromatographic separation was performed with mobile phases A (5 mM NH_4_AcO pH 9.9) and B (ACN) at a flow rate of 200 μL/min. A linear gradient from 15% A to 95% A over 18 min was followed by 7 min isocratic flow at 95% A and re-equilibration to 15% A. Metabolites were detected with a Thermo Scientific Q Exactive mass spectrometer run with polarity switching in full scan mode using a range of 70–975 *m/z* and 70.000 resolution. Maven (v 8.1.27.11) was used to quantify the targeted polar metabolites by AreaTop, using expected retention time and accurate mass measurements (< 5 ppm) for identification. Data analysis, including principal component analysis and heat map generation was performed using in-house R scripts. The normalized data represented is the metabolite intensity (area under the curve) normalized to the median intensity of all the metabolites in the respective sample.

### PQS quantification by thin layer chromatography (TLC)

Cultures were grown overnight at 37°C at 250 RPM. Afterwards, the strains were inoculated to an OD600 of 0.01 in 25 mL of fresh LB media supplemented with 30 ug/mL gentamicin and 0.2% arabinose. Cultures were grown for 8 hours at 37°C and shaking at 250 RPM reach peak PQS production in early stationary phase. 2 mL of culture were extracted using acidified ethyl acetate (0.1 mL acetic acid/1 L ethyl acetate) at a 1:1 ratio. The organic phase was removed and dried under nitrogen gas. Dried samples were resuspended using 15 μL of Optima grade methanol and 5 μL of samples were spotted onto a straight-phase phosphate-impregnated TLC plate after being activated for 1 hour at 100 °C. PQS standards (100 uM-500 μM) were also spotted on the same plate. After spotting, samples were allowed to run using a mobile phase consisting of 95:5 dichloromethane-methanol. PQS was visualized by intrinsic fluorescence after excitation using long-wave UV light. Digital images were captured and analyzed using the UVP, Inc., Gel Doc-It imaging system. PQS concentrations were determined using the VisionWorks LS Image Acquisition and Analysis software.

### Acute murine lung infection

12-week old male or female C57BL/6 mice were used for intratracheal infection experiments as described previously^26^. For the clinical isolate 216A, 1×10^7^ bacteria were intratracheally instilled per mouse but for acute infections of all other *P. aeruginosa* strains, 5×10^6^ bacteria were intratracheally instilled per mouse. At day 1 post infection, mice were euthanized by CO_2_ inhalation, and lung and liver tissues were harvested. The harvested tissues were homogenized in PBS and dilutions of the homogenates were plated on LB agar to enumerate viable bacteria. Lung homogenates were also used for RNA isolation and RT-qPCR to determine the expression of inflammatory genes.

### Chronic *Pa* lung infection in *Scnn1b*-Tg mice

The protocol by Facchini *et al.*^65^ was adapted to embed bacteria in SCFM2 agar beads. Briefly, strains were grown in synthetic CF sputum medium (SCFM2, Synthbiome Inc, USA) overnight at 37°C and shaking at 250 RPM. Overnight cultures were then diluted 1:400 in fresh SCFM2 and grown to mid-log phase at 37°C and shaking at 250 RPM. For *Pa* LESB58 pME6032 and pME6032::*mexEFoprN*, strains were grown in SCFM2 containing 200μg/mL tetracycline and 0.1mM IPTG. Bacterial cultures were pelleted and resuspended in 3 mL SCFM2 containing 1.5% agar. The bacterial-SCFM2 agar suspension was then added to prewarmed (50°C) heavy mineral oil and immediately stirred at RT for 6 minutes followed by another 30 minutes in iced water. The agar beads were washed in phosphate buffered saline (PBS) four times before final resuspension in PBS. The agar bead suspension was then homogenized, and serial dilutions of the homogenate were plated on LB agar to determine the volume of suspension required for infection.

For chronic infections, 12 week-old male or female *Scnn1b*-Tg mice were intratracheally instilled with 1×10^6^ bacteria embedded in SCFM2 agar beads. Mice were monitored for survival three times daily until day 7 post infection. At day 7 post infection or at earlier time points if mice were found to be moribund, mice were euthanized by CO_2_ inhalation, lungs were harvested in PBS and homogenized. Dilutions of the lung lysate were plated on LB agar to enumerate viable bacteria in the lungs.

### Flow cytometry

Infected mice were euthanized by CO_2_ inhalation. The lungs were perfused with PBS to flush out blood. Perfused lungs were then harvested, minced and digested using 0.2% collagenase II in RPMI containing 10% FBS. Red blood cells (RBC) were then removed from the digested tissue using RBC lysis buffer (Invitrogen, USA). 5×10^6^ cells from each sample were then resuspended in staining buffer (PBS+3% FBS+0.01% sodium azide) containing Fc block and LIVE/DEAD stain (Invitrogen, USA) and incubated at 4°C for 30 minutes. Cells were then washed and stained with the following antibodies: CD45, Siglec F, CD11b, CD11c, TCRβ, Ly6C and Ly6G (BD, USA) at 4°C in dark for 30 minutes. Fluorescence was then determined using a Cytek Aurora flow cytometer (Cytek biosciences, USA) and analyzed using the BD FlowJo software. The gating strategy for analysis has been depicted in Fig. S6.

### CF airway barrier dysfunction assay

Primary human epithelial cells from CF (ΔF508/ΔF508) airways were obtained under Cedars-Sinai Medical Center IRB approval STUDY00001735.Epithelial cells were cultured in PneumaCult-Ex Plus medium (Stemcell technologies, USA) on polystyrene trans-well inserts (pore size, 0.4μm) (Corning, USA). Once a confluent monolayer was established, cells were cultured at air-liquid interface (ALI) in PneumaCult-ALI medium (Stemcell technologies, USA) to induce differentiation into ciliated and mucous epithelial cells. The formation of tight junctions was confirmed by measuring the transepithelial electrical resistance (TEER) using STX2 chopstick electrodes (World Precision Instruments, USA). Since ALI cultures with TEER < 330 Ω.cm^2^ showed high permeability to 4kDa FITC dextran, only ALI cultures with TEER > 330 Ω.cm^2^ were used for the experiment.

To determine the effect of secreted virulence factor expression on CF airway barrier permeability, bacterial strains were first grown overnight in RPMI 1640 (Gibco, USA) containing 10% fetal bovine serum (Omega Scientific Inc, USA) at 37°C and shaking at 250 RPM. Cell free supernatants containing secreted virulence factors (bacterial supernatant) were harvested by filtering overnight cultures through a 0.22-μm syringe filter. The bacterial supernatants were then added to the apical surface of differentiated CF epithelia and integrity was microscopically observed every other hour until any of the treated samples showed epithelial monolayer defects. TEER of all the exposed CF epithelia were then measured to ascertain loss of barrier function. Further, at the end point, RPMI 1640 containing 4 kDa Fluorescein Iso-thiocyanate (FITC) labelled dextran was added to the apical surface and incubated for 60 minutes at 37°C, 5% CO_2_. The basal medium was then transferred to a 96-well clear bottom black plate to measure FITC fluorescence (Ex: 390 nm, Em: 420 nm) using a Varioskan Lux Microplate Reader (Thermo Fisher Scientific, USA).

### Statistics

All statistical analysis was performed using GraphPad Prism v10. All experiments were performed with at least three biological replicates and data has been represented as mean ± standard error of mean (SEM). Normality of variance was assessed by Shapiro-Wilk test. Student t-test or Mann-Whitney test was performed to analyze the significance between two unpaired groups based on data distribution. ANOVA followed by Tukey’s test and Kruskal-Wallis or Dunnett’s multiple comparisons tests, were performed for parametric and non-parametric analysis of significance between three or more groups. All testing was considered significant at the two-tailed p-value < 0.05.

## Supplementary Material

Supplement 1
