# Supplementary material for: Evolutionary loss of an antibiotic efflux pump increases Pseudomonas aeruginosa quorum sensing mediated virulence in vivo
Source: Res Sq. 2024 Nov 12:rs.3.rs-5391023. Preprint. [Version 1] doi: 10.21203/rs.3.rs-5391023/v1 (PMC11601840; doi:10.21203/rs.3.rs-5391023/v1)
Supplement: Supplement 1 [file NIHPPRS5391023V1-supplement-1.pdf]

Supplementary figure legends

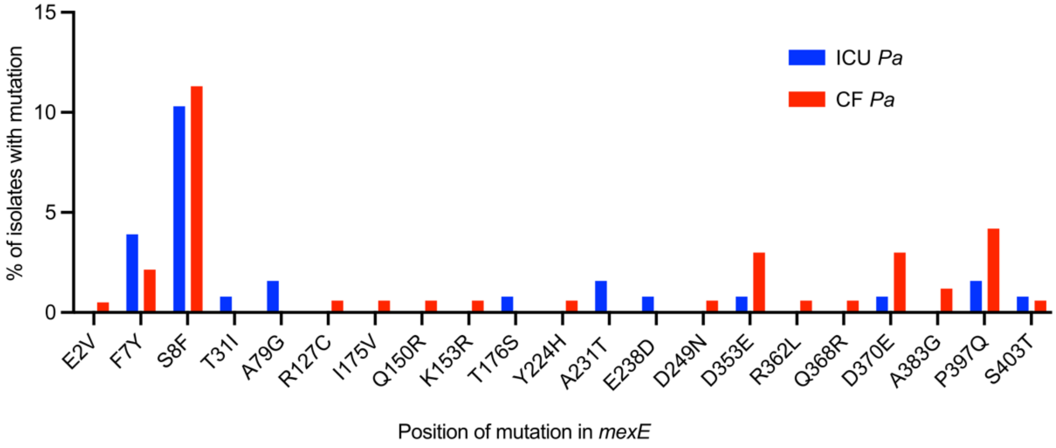

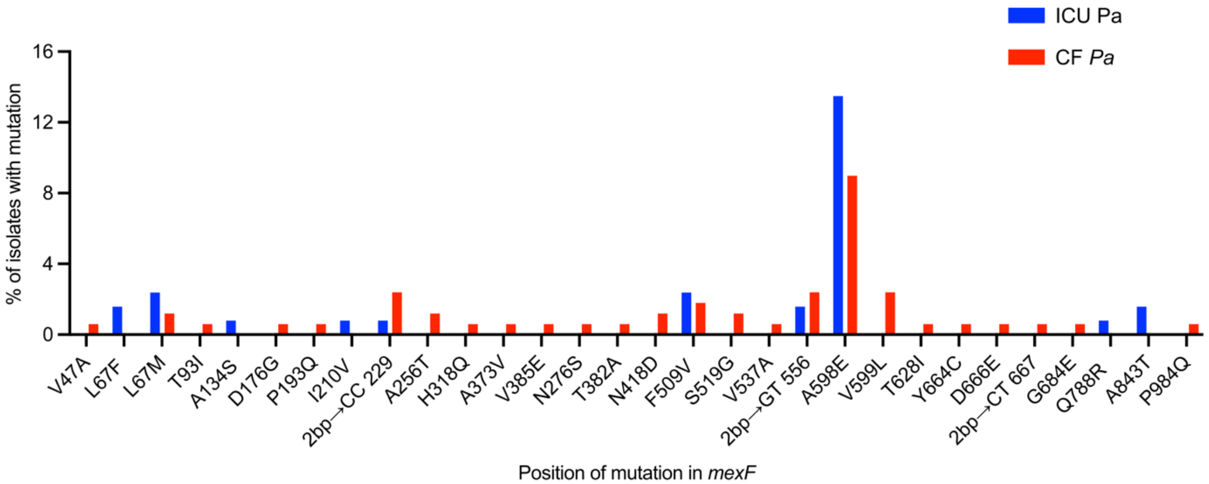

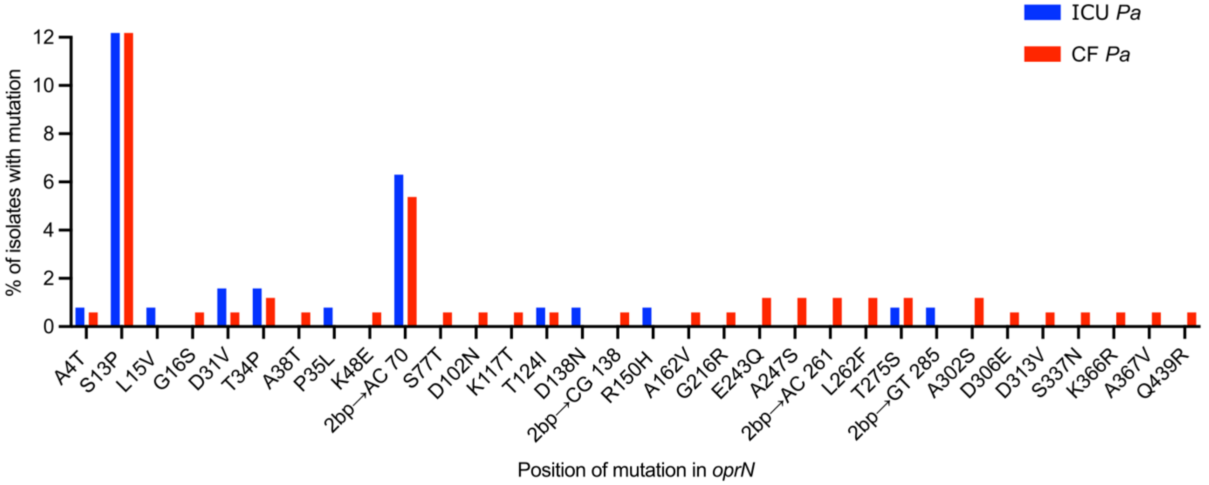

Fig. S1: Non-synonymous (NS) mutations in *mexE*, *mexF* and *oprN*. **a-c** Genomic location and prevalence of NS mutations in (a) *mexE*, (b) *mexF,* and (c) *oprN*. Mutations in ICU *Pa* respiratory isolates are represented in blue and mutations in CF *Pa* isolates are represented in red.

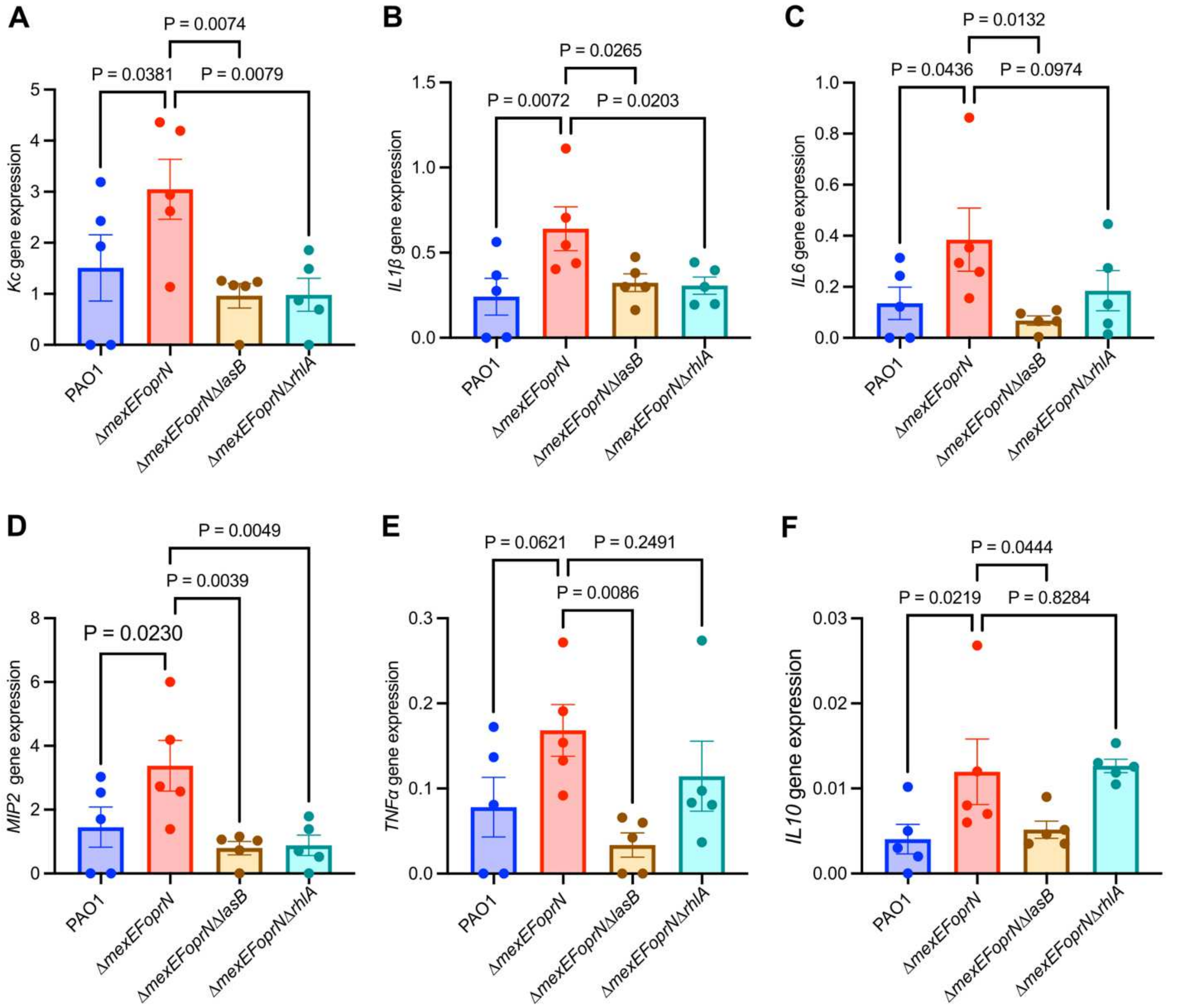

Fig. S2: PAO1 Δ*mexEFoprN* infection causes increased lung inflammation. a-f RT-qPCR for gene expression of inflammatory cytokines *Kc*, *Il1β*, *Il6, Mip2*, *Tnfα* and *Il10* in the lung tissue of PAO1, PAO1 Δ*mexEFoprN,* PAO1 Δ*mexEFoprN*Δ*lasB* or PAO1 Δ*mexEFoprN*Δ*rhlA* infected C57BL/6 mice at 24 hpi. Gene expression was normalized to *ppia*. Data show mean ± SEM. Statistical significance analyzed by ANOVA.

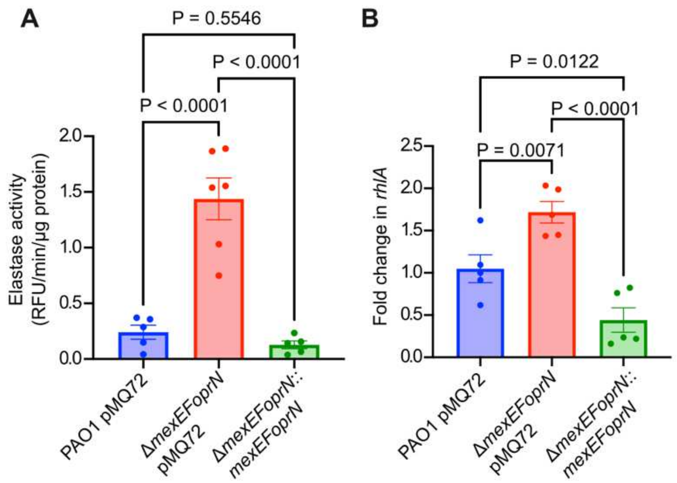

Fig. S3: Complementation reduces elastase and *rhlA* levels in PAO1 Δ*mexEFoprN.* **a** Elastase activity determined from the supernatants of PAO1 pMQ72, PAO1 Δ*mexEFoprN* pMQ72 and PAO1 Δ*mexEFoprN* pMQ72::*mexEFOprN*. **b** Fold change in *rhlA* gene expression in PAO1 pMQ72, PAO1 Δ*mexEFoprN* pMQ72 and PAO1 Δ*mexEFoprN* pMQ72::*mexEFoprN* measured by RT-qPCR. Gene expression was normalized to *rpoD*. Data shown mean± SEM and statistical significance was analyzed by ANOVA.

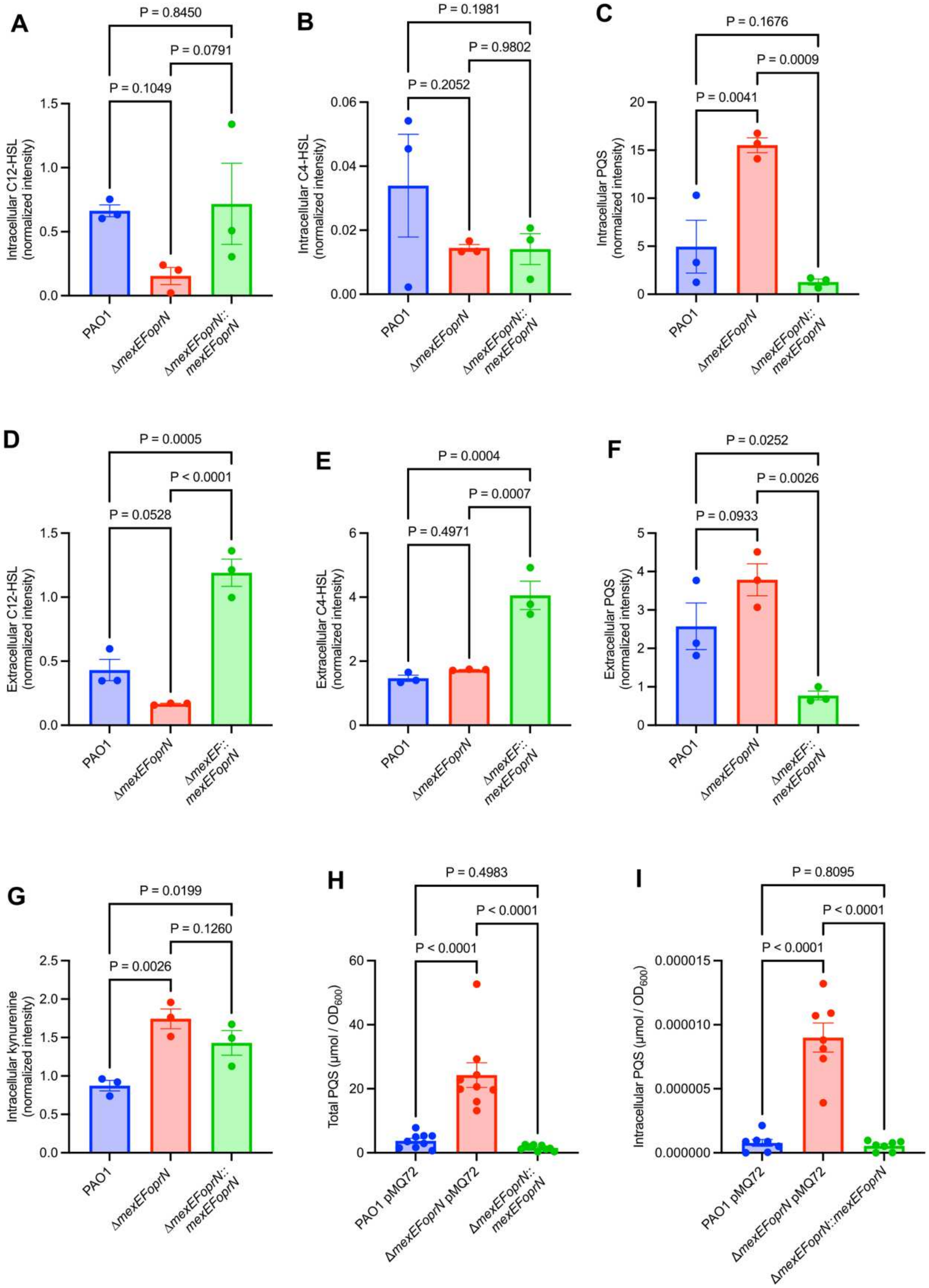

Fig. S4: PQS and its precursor kynurenine are more abundant in PAO1Δ*mexEFoprN*. **a-c** Intracellular levels of C12-HSL, C4-HSL and PQS in PAO1 pMQ72, PAO1 Δ*mexEFoprN* pMQ72 and PAO1 Δ*mexEFoprN* pMQ72::*mexEFOprN* analyzed by mass spectrometry.

d-f Extracellular levels of C12-HSL, C4-HSL and PQS in PAO1 pMQ72, PAO1 Δ*mexEFoprN* pMQ72 and PAO1 Δ*mexEFoprN* pMQ72::*mexEFOprN* analyzed by mass spectrometry. **g** Intracellular levels of kynurenine in PAO1 pMQ72, PAO1 Δ*mexEFoprN* pMQ72 and PAO1 Δ*mexEFoprN* pMQ72::*mexEFOprN* analyzed by mass spectrometry. **h-i** Total and intracellular levels of PQS in PAO1 pMQ72, PAO1 Δ*mexEFoprN* pMQ72 and PAO1 Δ*mexEFoprN* pMQ72::*mexEFOprN* estimated by thin layer chromatography, respectively. Data show mean ± SEM and statistical significance was analyzed by ANOVA.

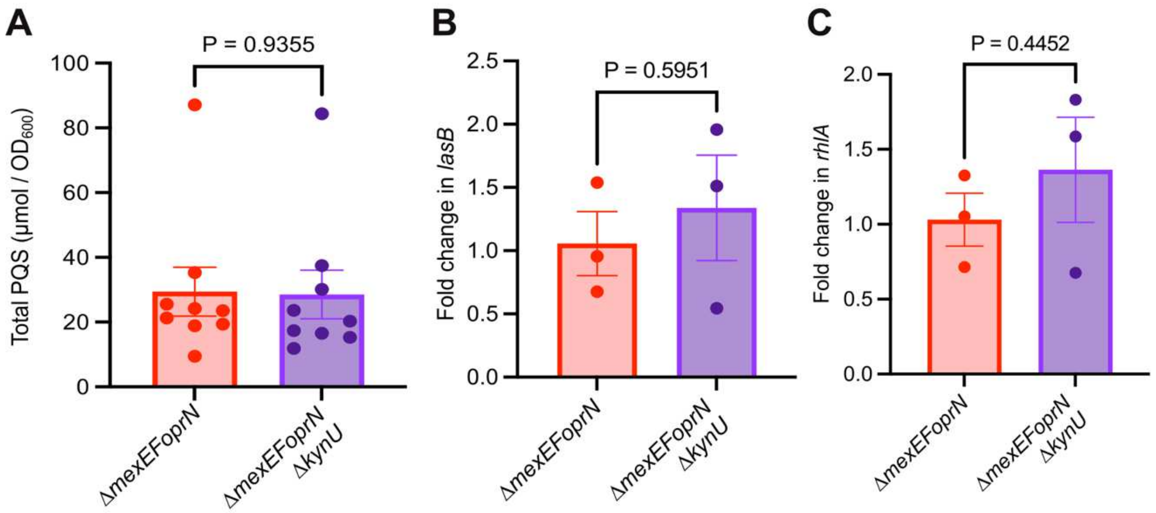

Fig. S5: Kynurenine biosynthesis is not required for increased PQS or virulence gene expression in PAO1 Δ*mexEFoprN*. a Total levels of PQS in PAO1 Δ*mexEFoprN* and PAO1 Δ*mexEFoprN*Δ*kynU* quantified by TLC. **b** Fold change in *lasB* gene expression in PAO1 Δ*mexEFoprN* and PAO1 Δ*mexEFoprN*Δ*kynU* measured by RT-qPCR*.* Gene expression was normalized to *rpoD*. **c** Fold change in *rhlA* gene expression in PAO1 Δ*mexEFoprN* and PAO1 Δ*mexEFoprN*Δ*kynU* measured by RT-qPCR*.* Gene expression was normalized to *rpoD*. Data shown mean ± SEM and statistical significance analyzed by unpaired t-test.

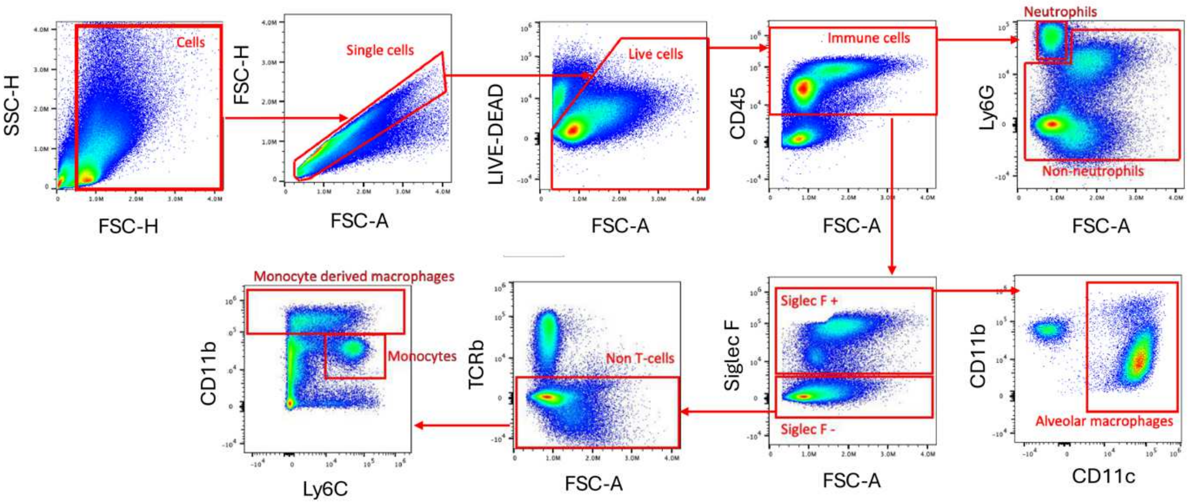

Fig. S6: Gating strategy for flow cytometry analysis of immune cells in the lungs of infected mice. Live single cells were identified using a LIVE-DEAD stain. Single cells expressing CD45 were identified as immune cells. Ly6G+ immune cells were identified as neutrophils. SiglecF+CD11b+CD11c+ immune cells were identified as alveolar macrophages. SiglecF- immune cells hat did not express TCRb receptors were further classified as monocytes ($Ly6C^{hi}CD11b^{lo}$) or monocyte derived macrophages ($Ly6C^{lo}CD11b^{hi}$).

Supplementary tables

Table S1. Differentially expressed genes between PAO1 and PAO1 ∆*mexEFoprN*.

Table S2. List of strains used in this study.

Table S3. List of primers used in this study.

Supplementary Files

This is a list of supplementary files associated with this preprint. Click to download.

- TableS1.xlsx
- TableS2.xlsx
- TableS3.xlsx
